# Personal

**Published:** 1909-09

**Authors:** 


### PERSONAL.

---

Dr. Edward T. Abrams, of Dollar Bay, was elected president of the Upper Peninsula (Michigan) Medical Society, at its annual meeting held at Calumet, August 4, 1909. The next meeting (1910) will be held at Sault Ste. Marie.

DR. ALFRED H. NOEHREN, of Buffalo, announces the removal of his office to 644 Kensington Avenue, corner Grider Street, opposite Water Tower. Hours: 8 to 9 a. m., 1 to 3 and 7 p. m. Branch office, 571 E. Utica Street. Telephone Crescent 274-R.

---

DR. JOHN HUDSON GRANT, of Delaware Avenue, Buffalo, sailed August 14th for London, to remain abroad for a few weeks.

---

DR. GEORGE H. HIMMELSBACH, of Buffalo, took steamer for Europe about the middle of August. He expects to return and take up his work in the early autumn.

